# Characterizing the Role of Orco Gene in Detecting Aggregation Pheromone and Food Resources in *Protaetia brevitarsis* Leiws (Coleoptera: Scarabaeidae)

**DOI:** 10.3389/fphys.2021.649590

**Published:** 2021-04-13

**Authors:** Xiaofang Zhang, Panjing Liu, Qiuju Qin, Min Li, Runjie Meng, Tao Zhang

**Affiliations:** ^1^Institute of Plant Protection, Hebei Academy of Agriculture and Forestry Sciences, Integrated Pest Management Center of Hebei Province, Baoding, China; ^2^Key Laboratory of IPM on Crops in Northern Region of North China, Ministry of Agriculture, Baoding, China; ^3^College of Plant Protection, Hebei Agricultural University, Baoding, China; ^4^Baoding Vocational and Technical College, Baoding, China

**Keywords:** *Protaetia brevitarsis*, olfactory recognition, semiochemicals, host seeking, RNAi

## Abstract

An accurate olfactory system for recognizing semiochemicals and environmental chemical signals plays crucial roles in survival and reproduction of insects. Among all olfaction-related proteins, olfactory receptors (ORs) contribute to the conversion of chemical stimuli to electric signals and thereby are vital in odorant recognition. Olfactory receptor co-receptor (Orco), one of the most conserved ORs, is extremely essential in recognizing odorants through forming a ligand-gated ion channel complex with conventional ligand-binding odorant receptors. We have previously identified aggregation pheromone in *Protaetia brevitarsis* (Coleoptera: Scarabaeidae), a native agricultural and horticultural pest in East-Asia. However, to our best knowledge, its olfaction recognition mechanisms are still veiled. To illustrate how *P. brevitarsis* recognize aggregation pheromone and host plants, in the present study we cloned and sequenced the full-length *Orco* gene from *P. brevitarsis* antennae (named *PbreOrco*) and found that *PbreOrco* is highly conserved and similar to Orcos from other Coleoptera insects. Our real-time quantitative PCR (qRT-PCR) results showed that *PbreOrco* is mainly expressed in antenna. We also demonstrated that silencing *PbreOrco* using RNA interference through injecting dsOrco fragment significantly inhibited *PbreOrco* expression in comparison with injecting control dsGFP and subsequently revealed using electroantennogram and behavioral bioassays that decreasing *PbreOrco* transcript abundance significantly impaired the responses of *P. brevitarsis* to intraspecific aggregation pheromone and prolonged the time of *P. brevitarsis* spending on food seeking. Overall, our results demonstrated that *PbreOrco* is crucial in mediating odorant perception in *P. brevitarsis*.

## Introduction

Having a precise olfactory system is of great benefit for most insects in foraging, mating, locating oviposition sites and avoiding adverse environments (Leal, 2013; Brito et al., 2016). Olfactory recognition is a complicated and sophisticated process involving numerous receptors and signaling pathways. The odorant stimuli are firstly detected by the olfactory receptor neurons (ORNs) in insect antennae and processed to bioelectric signals, which are subsequently transmitted to the main nervous system (e.g., brain), inducing various odor-evoked behaviors (Leal, 2013; Fleischer et al., 2018). During the odorants processing in ORNs, an acceptable hypothesis infers that odorants are specifically transported by odorant binding proteins (OBPs) and chemosensory proteins (CSPs) to the olfactory receptors (ORs), which belong to a family of seven-transmembrane domain proteins on the dendrite membrane of neurons and are conceived to be essential in odorant recognition (Smart et al., 2008), and thereby recognized and converted to electric signals and subsequently degraded by odorant degrading enzymes (ODEs) (Sato and Touhara, 2008; Zhou, 2010; Leal, 2013).

To recognize chemical signals, most insect ORNs express two subclasses of ORs—a conventional odorant-specific olfactory receptors and a highly conserved olfactory receptor co-receptor (Orco) (Harini and Sowdhamini, 2012). Compared to conventional ORs, Orco is more conserved (Missbach et al., 2014; Lin et al., 2015) among a variety of arthropods including Lepidoptera, Coleoptera, Hymenoptera, Orthoptera, Hemiptera and Diptera (Krieger et al., 2003; Yang et al., 2012; Lin et al., 2015; Li et al., 2016; Wang et al., 2018). During the process of recognizing odorants, Orco couples with conventional ORs to form an Orco-ORx complex, which functions as a ligand-gated ion channel and determines the sensitivity and specificity of the ORN where it is expressed (Breer et al., 2019). In this complex, Orco is a key factor for the localization, stability and correct protein folding of ORs (Larsson et al., 2004; Stengl and Funk, 2013). Studies have shown that knockout or mutation of *Orco* gene would lead to the disablement of odorant sensing in insects (Larsson et al., 2004; Neuhaus et al., 2005). For examples, *Orco* mutations in fruit flies, locusts, mosquitoes and moths lead to loss of OR function, and impaired responses to odorants such as food volatiles and sex pheromones (Asahina et al., 2008; DeGennaro et al., 2013; Koutroumpa et al., 2016; Li et al., 2016; Yang et al., 2016; Trible et al., 2017), silencing of *Orco* through RNA interference (RNAi) in beetles (*Tenebrio molitor*, *Dendroctonus armandi*, and *Ophraella communa*) impairs their ability to locate hosts and mates (Liu et al., 2016; Zhang et al., 2016; Ma et al., 2020). In addition, Orco is also involved in other important physiological activities (such as wing differentiation, metabolism regulation, stress resistance, number of glomeruli in antennal lobes and life span extension), indicating they may also participate in more physiological functions (Libert et al., 2007; Fan et al., 2015; Trible et al., 2017).

The white-spotted flower chafer (WSFC), *Protaetia brevitarsis* Leiws (Coleoptera: Scarabaeidae), is a native agricultural and horticultural pest in East-Asia, including China, Korean Peninsula, Japan, Thailand, Mongolia and Russia (Suo et al., 2015; Liu et al., 2019). WSFC larvae, which feed on soil humus, decaying plant residues, and even fermented animal manure, are cultivated as a potential resource insect for converting herbaceous and plant residues to organic fertilizer (Li et al., 2019; Wang et al., 2019). However, WSFC adults are destructive to many important crops, such as corn, wheat, apple, peach and various vegetables (Zhao and Chen, 2008; Xu et al., 2009; Cai et al., 2020). To environmentally-friendly control and monitor WSFC, we have identified 4-methylanisole (4-MA) as an aggregation pheromone for developing efficient lures (Zhang et al., 2019). Although its candidate chemosensory receptors have been identified (Liu et al., 2019), the molecular mechanisms underlying olfactory recognition, including function of *PbreORs*, remain largely unexplored. Previously, based on transcriptome analysis, we identified a *PbreOrco*-related sequence encoding a 288aa peptide, though the 5′ terminus was suspected to be missing.

To fully explore the functions of *PbreOrco*, in this study, we firstly cloned the full-length sequence of *PbreOrco* through rapid amplification of cDNA ends (RACE), analyzed its characteristics and expression pattern. We then silenced *PbreOrco* gene using RNAi and examined its pheromone- and food-seeking function. Our results could further deepen our understanding on Orco functions and benefit subsequent development of semiochemical-based strategy to control this pest.

## Materials and Methods

### Insect Rearing and Tissue Collection

White-spotted flower chafer larvae were reared on fermented wheat straw in a constant environment with temperature of 25 ± 2°C, relative humidity of 50 ± 2% and photoperiod of 14L:10D (Liu et al., 2019). Newly-emerged adults were sorted by sex and fed with fresh peach. When unmated WSFCs reached approximately 7 days old, their antennae, head without antennae, thorax, abdomen, legs and wings were excised, immediately frozen in liquid nitrogen, and stored at −80°C for future experiments.

### Total RNA Extraction and cDNA Synthesis

Total RNA was extracted from 50 WSFCs of each sex using TRIzol reagent (TransGen, China) following the manufacturer’s instructions. RNA quality was evaluated by 1.0% agarose gel electrophoresis and Nanodrop 2000 (OD_260_/OD_280_ ranged from 1.80 to 2.10). The first-strand cDNA was synthesized from 1 μg of total RNA using All-in-One First-Strand cDNA Synthesis SuperMix (TransGen, China) according to the manufacturer’s instructions. The synthesized cDNA was stored at −20°C prior to further analysis.

### Rapid Amplification of cDNA Ends to Obtain Full-Length *PbreOrco* Gene

According to the reported incomplete *PbreOrco* sequence (MH324899), the 5′ end of mRNA was obtained using a 5′-RACE Kit (Sangon, China) with the gene-specific primers (GSP1 and GSP2) listed in Supplementary Table 1 following the manufacturer’s protocol. Briefly, the first strand cDNA was synthesized by using specific reverse transcription primers (5′ RACE-RT Primer, Supplementary Table 1) and reverse transcriptase mix (RNase H-). Two rounds of touchdown PCR was performed as follows: 94°C for 1 min; 10 cycles of 94°C for 60 s, 70°C (each cycle descends 1°C) for 30 s and 72°C for 60 s; 25 cycles of 94°C for 60 s, 60°C for 30 s, and 72°C for 60 s; and a final incubation at 72°C for 10 min. The first round of PCR amplification was carried out with GSP1 as downstream primer and the first strand of cDNA as template. Then, the 5′ end cDNA of *PbreOrco* was amplified by using a universal 5′ RACE outer primer containing partial splice sequence (Supplementary Table 1) as the upstream primer and the GSP2 as the downstream primer. The PCR product was purified, ligated into a pEASY-Blunt vector (TransGen Biotech, China), and sequenced (Sangon Biotech, China).

### Sequence Analysis

The homology of PbreORCO protein was analyzed by Blastp search in NCBI database^1^. Its transmembrane domains were identified using the TMHMM Server v. 2.0 program^2^. Its topology diagram was constructed using the TOPO2 Transmembrane Protein Display^3^. Protein sequences alignment was performed using ClustalX 1.83, and the results were presented by GeneDoc 2.7.0 software. Evolutionary analyses were conducted in MEGA7 using the Maximum Likelihood method with a bootstrap test (1000 replicates, complete deletion, NN) (Kumar et al., 2016). The evolutionary distances were computed using the Poisson correction model (Zuckerkandl and Pauling, 1965). When the number of common sites was < 100 or less than one fourth of the total number of sites, the maximum parsimony method was used; otherwise BIONJ method with MCL distance matrix was used. Finally, phylogenetic trees were viewed and edited using FigTree v.1.4.3^4^. Identity calculation of Orcos in various insects was analyzed using MegAlign (DNAStar Lasergene 12.1) with the pair distances of Untitled ClustalW (slow/accurate, identity).

### Tissue Expression Profiles of *PbreOrco*

The expression of *PbreOrco* in different tissues was analyzed using real-time quantitative PCR (qRT-PCR) with an ABI 7500 Real-Time PCR System (Applied Biosystems, United States). The primers for *PbreOrco* was designed by Primer 6.0 (Supplementary Table 1). GADPH2 was used as reference gene according to our previous study (Liu et al., 2019). qRT-PCR reactions were performed in 20 μL reaction mixtures, each containing 10 μL TransStart Tips Green Mix (TransGen, China), 0.5 μL of each primer (10 μM), 1 μL of sample cDNA, and 8 μL of sterilized H_2_O. The thermocycling conditions were as follows: 95°C for 3 min; 40 cycles at 95°C for 10 s; and an annealing temperature of 60°C for 30 s. Each test was carried out three times as technical replicates. The amplification efficiency of the primers was 92–98% according to standard curve analysis. Relative expression of *PbreOrco* were analyzed using the 2^–ΔΔCT^ method (Livak and Schmittgen, 2001). Three independent biological repeats were conducted, and each RNA sample was extracted from 30 adults.

### RNAi of *PbreOrco* Gene and qRT-PCR Validation

The fragment of *PbreOrco* was amplified using specific primers with T7 RNA polymerase promoter (Supplementary Table 1). A double-stranded green fluorescent protein (dsGFP) fragment amplified from the *GFP* gene (GenBank No. U50963) was used as the negative control. Double-stranded RNA (dsRNA) was synthesized using the T7 Ribomax Express RNAi System (Promega, Madison, WI, United States). The quality and concentration of dsRNA were determined by agarose gel electrophoresis and Nanodrop 2000 (Thermo, United States). The newly emerged WSFCs were separated and reared individually before dsRNA injection. To each beetle, 3 μg of dsOrco or dsGFP was injected into the conjunction between the head and thorax using a microsyringe. The antennae were collected at 1, 3, 5, 7, and 9 days of post-injection to evaluate the expression of *PbreOrco* using qRT-PCR. Three independent biological repeats were collected, and each repeat contained 30 adults.

### Electroantennography

The electrophysiological responses of injected WSFCs to the aggregation pheromone 4-MA (99% purity, Aladdin Reagent Co., Ltd., Shanghai China) were monitored on an Electroantennography (EAG) apparatus (Syntech Ltd., Kirchzarten, Germany) following a reported method (Zhang et al., 2020). Briefly, the antennae of WSFCs at 6–7 days of post-injection prepared by cutting off the tips were affixed to the recording electrode with electrically conductive gel and flowed over by a constant humidified airstream (∼200 mL⋅min-1). After that, a pulse airstream carrying volatiles from 20 μg of 4-MA in 20 μL paraffin oil was brought to the antennae through constant airstream at 30 s intervals by an air stimulus controller (CS-55). The electric signals generated by the responses of antennae were recorded and analyzed using the Syntech EAG 2000 software (Syntech, Kirchzarten, Germany). Paraffin oil was used as parallel solvent control. The EAG responses to pheromone of each treatment were calibrated by subtracting the EAG values to solvent control. Five antennae were tested with five stimuli for each antenna.

### Insect Behavioral Bioassay

The responses of female and male WSFCs at 6–7 days of post-injection to 4-MA were tested by a two-choice bioassay using a glass Y-tube olfactometer (3.0 cm inner diameter) comprised of a 25-cm stem and two 20-cm branching arms at an angle of about 60° (Ikeura et al., 2012). During the assay, charcoal-filtered and humidified air was pumped through the olfactometer at a rate of 100 ml⋅min-1 using an atmosphere sampling instrument (QC-1B, Beijing Municipal Institute of Labor Protection, Beijing, China) and 20 μl of odor sources (0.1 μg/μL in paraffin) on strips of filter paper (1 cm × 5 cm) were put into sample bottles connected to the branching arms and an injected WSFC individual was released into the stem (Yang et al., 2017). The 4-MA in paraffin oil was placed into one arm of the Y-tube, while paraffin oil was placed into the other arm as the negative control. Y-tube was cleaned with ethanol after each test. Each experiment contained 30 injected adults and lasted for 20 min.

For food-seeking behavior, the injected WSFCs (pre-starved for 24 h) were released into the four corners of a transparent box. After the insects adapted to the environment (∼15 min), half of a fresh peach was introduced into the center of the box. The WSFCs were allowed to seek food for 20 min, during which the number of WSFCs arrived food and their foraging time were recorded. If an insect has failed in arriving the food within 20 min, it shall be judged unable to seek food (Liu et al., 2016). Three independent biological repeats were conducted, and each experiment contained 10 post-injected adults.

### Statistical Analysis

To analyze the results of qRT-PCR, EAG tests, Y-tube tests and food seeking behavior, one-way analysis of variance (ANOVA) with Tukey’s test was used in SPSS 19.0 software. The least significant significance was set at *P* < 0.05.

## Results

### Identification of Full-Length PbreOrco

**FIGURE 1:**
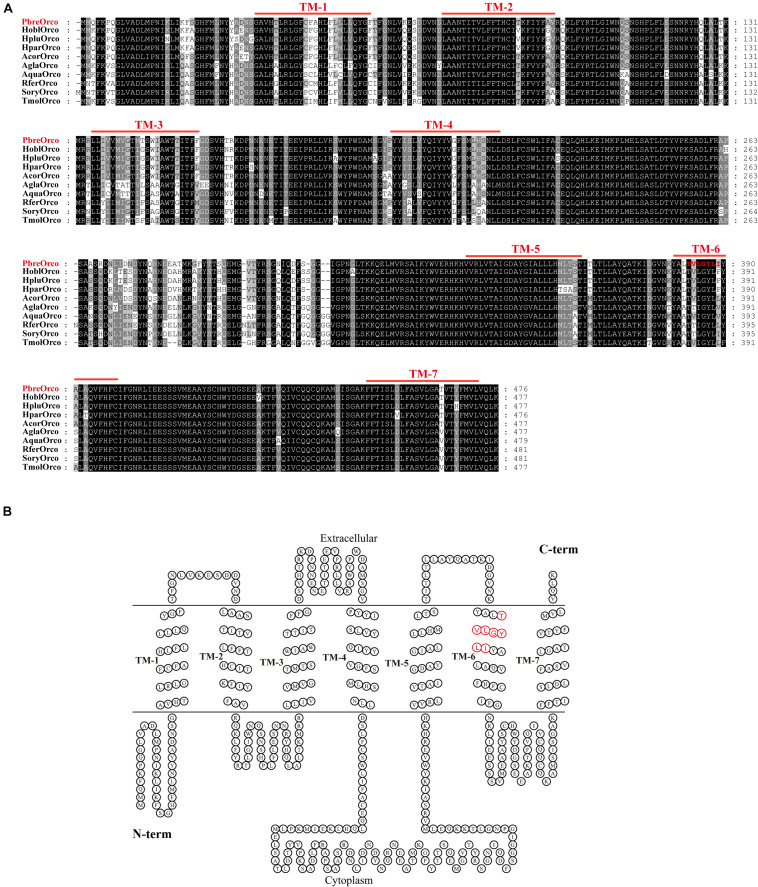
Sequence analysis of *PbreOrco*. **(A)** Amino acid sequence alignment of *PbreOrco* with orther Orcos from Coleoptera insects. Hpar, Holotrichia parallela; Hobl, Holotrichia oblita; Hplu, Holotrichia plumbea; Acor, Anomala corpulenta; Agla, Anoplophora glabripennis; Aqua, Ambrostoma quadriimpressum; Rfer, Rhynchophorus ferrugineus; Sory, Sitophilus oryzae; Tmol, Tenebrio molitor. The sequences used in this analysis listed in Supplementary Table 2. **(B)** Seven-transmembrane topology of representative *PbreOrco*. The double line represents the membrane region with labeled extracellular and cytoplasmic sides. TM: transmembrane. The conserved motif (383–389: TVLGYLI) was displayed in red.

The full-length sequence of *PbreOrco* was obtained based on the reported sequence (MH324899) using 5′-RACE and submitted to GenBank (Access No: MW382164). The open reading frame (ORF) of *PbreOrco* was 1,431 bp and encodes a protein comprising 476 amino acids. The transmembrane prediction results indicated that *PbreOrco* has seven transmembrane domains with an intracellular N-terminus and an extracellular C-terminus, indicating it is a typical Orco protein (Figure 1).

### Sequence Analysis of *PbreOrco*

Increasing reports demonstrate that Orco receptors are highly conserved during insect evolution. Sequence alignment of *PbreOrco* with Orco from nine other Coleoptera insects (*Holotrichia parallela*, *H. oblita*, *H. plumbea*, *Anomala corpulenta*, *Anoplophora glabripennis*, *Ambrostoma quadriimpressum*, *Rhynchophorus ferrugineus*, *Sitophilus oryzae*, and *Tenebrio molitor*) revealed a relatively high amino acid identity. *PbreOrco* was 91.19, 91.19, 91.39, 92.24, 80.71, 79.96, 80.28, 79.42, and 81.21%, respectively, homologous with *HoblOrco* (*H. oblita*), *HpluOrco* (*H. plumbea*), *HparOrco* (*H. parallela*), *AcorOrco* (*A. corpulenta*), *AglaOrco* (*A. glabripennis*), *AquaOrco* (*A. quadriimpressum*), *RferOrco* (*R. ferrugineus*), *SoryOrco* (*S. oryzae*), and *TmolOrco* (*T. molitor*). In addition, the C-terminal sequences (TM5-TM7) were highly conserved (Figure 1).

**FIGURE 2:**
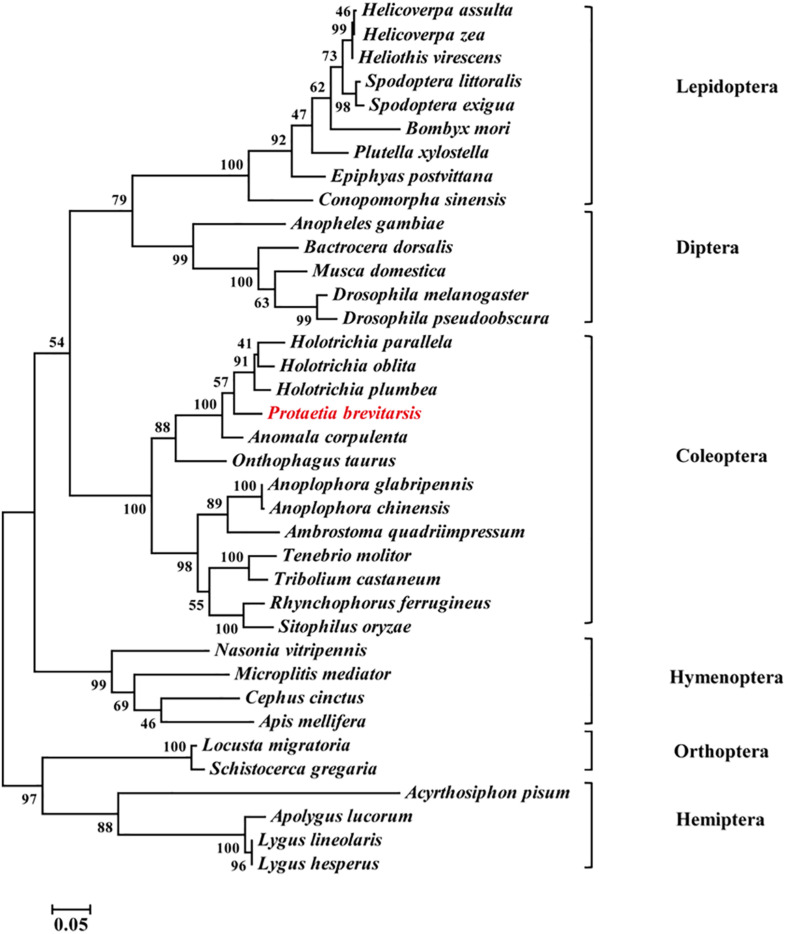
Phylogenetic analysis of Orco orthologs from 37 insect species. The branch lengths were proportional to the percentage of sequence difference (scale: 0.05% difference). Bootstrap values expressed as percentages of 1,000 replications are shown at branch nodes. The *PbreOrco* sequence was shown in red. The sequences used in this analysis are listed in Supplementary Table 2.

Thirty-seven Orco sequences from six insect orders were used to construct a phylogenetic tree. The phylogenetic analysis showed that Coleoptera, Lepidoptera, Diptera and Hymenoptera were clustered together in a large branch, while Orthoptera and Hemiptera were in another branch. Compared with other insect Orcos, *PbreOrco* presented a close relationship with Orco of Coleoptera (Figure 2).

### Expression Profiles of *PbreOrco*

**FIGURE 3:**
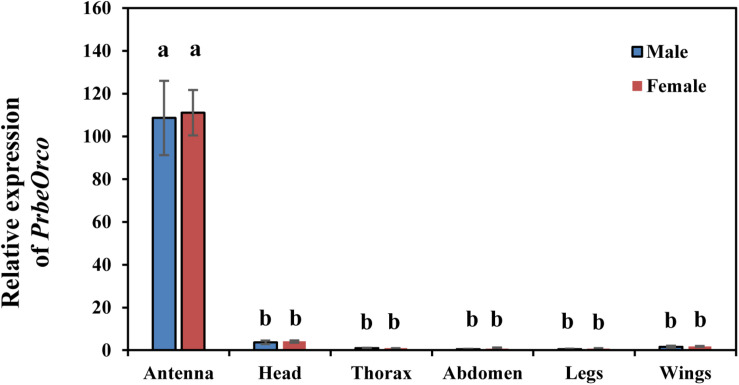
Expression profiles of *PbreOrco* in different tissues of male and female. The head excluded antennae and maxillary palps. The relative expression levels were normalized to the GADPH2 gene, with the expression of male thorax as the calibrator. Different letters represent significant difference (*P* < 0.05).

qRT-PCR was used to determine the relative expression of *PbreOrco* in different adult tissues. The results showed that the expressions of *PbreOrco* in the antennae of both male and female WSFCs were significantly higher than those in other tissues. Furthermore, there was no significant difference in the expression of Orco between male and female antennae (Figure 3).

### RNAi Efficiency

**FIGURE 4:**
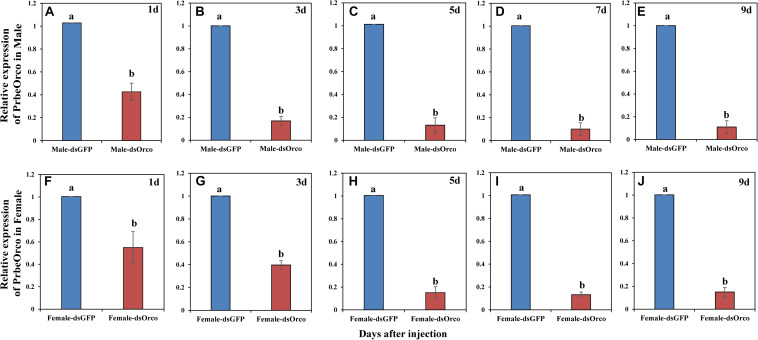
Relative expression level of *PbreOrco* after dsRNA injection. **(A–E)** Relative expression of *PrbeOrco* in male. **(F–J)** Relative expression of *PrbeOrco* in female. The relative expression levels were normalized to the GADPH2 gene. And the expression of the corresponding dsGFP-injected as the calibrator in each image. Different letters represent significant difference (*P* < 0.05).

During our experiment, the injected WSFCs were all alive. The qRT-PCR results showed that injecting dsRNA significantly decreased the expression of *PbreOrco*. Compared to the dsGFP-injected control WSFCs, the expression of *PbreOrco* was significantly inhibited at 1–9 days of post-injection dsOrco (Figure 4). The knockdown rates were 86.07 and 85.04% for male and female at 5 days of post-injection, and maintained at > 85% in the following several days (Figure 4). Consequently, WSFCs at 6–8 days of post-injection were selected for electrophysiological and behavioral bioassays.

### Silencing *PbreOrco* Impairs the Response to Aggregation Pheromone

**FIGURE 5:**
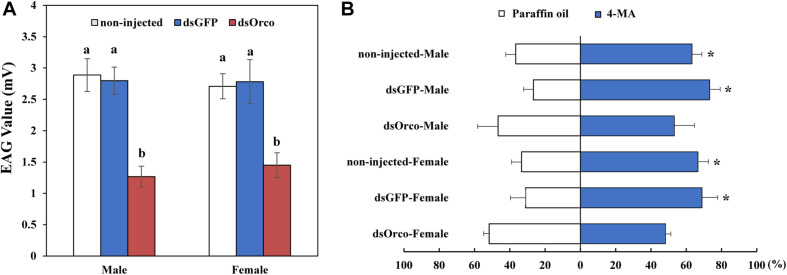
Responses of *P. brevitarsis* to aggregation pheromone, 4-methylanisole (4-MA). **(A)** Electroantennographic (EAG) responses of dsOrco- injected dsGFP-injected and non-injected *P. brevitarsis* to 4-MA. **(B)** Behavioral response of *P. brevitarsis* to 4-MA in a Y-tube olfactometer. Different letters represent significant difference (*P* < 0.05).

Electroantennography and olfactometer assays were performed to compare the responses of dsGFP-injected and dsOrco-injected WSFCs to aggregation pheromone. The average response values of dsOrco-injected WSFCs nearly halved in comparison with those of dsGFP-injected WSFCs (males: 2.80 mV ± 0.22 vs 1.27 mV ± 0.17 mV; females: 2.78 mV ± 0.35 vs 1.45 mV ± 0.20 mV, respectively) (Figure 5A and Supplementary Figure 1). The subsequent olfactometer assays showed that the dsOrco-injected WSFCs showed no preference to 4-MA or solvent control (Male: *F_1,4_* = 0.500, *P* = 0.519; Female: *F_1,4_* = 1.997, *P* = 0.230), while non-injected (Male: 65.56 ± 5.09%; *F_1,4_* = 32.00, *P* = 0.005; Female: 71.48 ± 5.70%; *F_1,4_* = 50.00, *P* = 0.002) and dsGFP-injected WSFCs (Male: 73.33 ± 5.77%; *F_1,4_* = 98.00, *P* = 0.001; Female: 68.96 ± 8.91%; *F_1,4_* = 28.016, *P* = 0.006) significantly moved toward 4-MA (Figure 5B), further confirming that RNAi-based silencing of *PbreOrco* impaired the response of both female and male WSFCs to aggregation pheromone.

### Silencing *PbreOrco* Influnces Food Seeking

**FIGURE 6:**
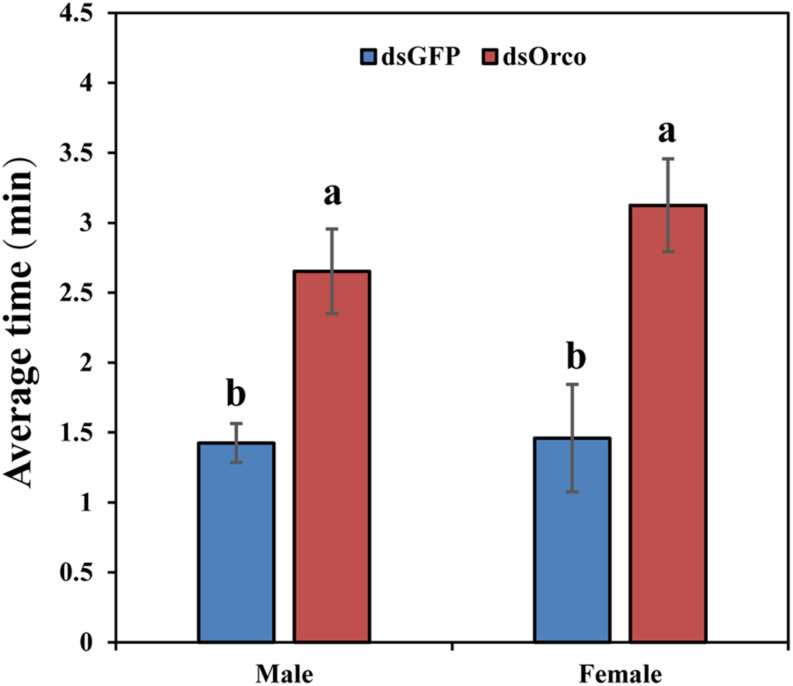
Time required for food searching in dsGFP- and dsOrco-injected *P. brevitarsis*. Different letters represent significant difference (*P* < 0.05).

To further verify the function of *PbreOrco* in insect feeding behavior, we set up behavioral experiments to test the food-seeking activity in response to fresh peaches. All the tested WSFCs had been starved for 24 h prior to the bioassays. The results showed that only 48.52% (male) and 46.67% (female) insects successfully arrived food within 20 min, significantly lower than dsGFP-injected (male: 76.67%; female: 79.63%) (Supplementary Figure 2). Furthermore, compared with the dsGFP-injected WSFCs, the dsOrco-injected took more time to find food during the test time (male: 2.65 vs 1.43 min; female: 3.13 vs 1.46 min) (Figure 6 and Supplementary Video 1).

## Discussion

Odorant receptors (ORs), which transform volatile stimuli to electrical signals in olfactory of insects, play important roles in recognition of various odorants (Leal, 2013). Among all ORs, Orco is the most particular and essential one: it assists the specific-ORs to bind and recognize odorants by forming a heteromeric OR-Orco complex rather than responding to odorants directly (Stengl and Funk, 2013). Thus, identification and functional study of insect Orco could provide further insights into function of ORs. In this study, we successfully cloned the full-length sequence of *PbreOrco* using the 5′RACE system from the antennae of *P. brevitarsis* and demonstrated the crucial role of *PbreOrco* in the olfactory mechanism of *P. brevitarsis*.

An abundance of reports have documented that insect Orcos are highly conserved amongst species (Krieger et al., 2003; Jones et al., 2005; Yang et al., 2012). As expected, the alignment and homology analysis showed that the sequence of *PbreOrco* is highly conserved with its orthologs in other beetles (Figure 1A). Especially, its C-terminus showed extremely high conservation among species. This region has been demonstrated to play an indispensable role in the functional interaction of the OR and Orco proteins (Hopf et al., 2015; Butterwick et al., 2018). Furthermore, motif TVVGYLG (393–399) located at the sixth predicted transmembrane helix of *DmelOrco* in *Drosophila melanogaster* was thought to comprise a ligand-gated selectivity filter with Val394 and Leu398 in the pores of K^+^ channels (Wicher et al., 2008). In *PbreOrco* sequence, a motif (383–389: TVLGYLI) (Figure 1), which is also located in the sixth transmembrane helix, is similar to the motif TVVGYLG in *DmelOrco*, indicating *PbreOrco* might function *via* the same mechanism. In addition, qRT-PCR determination showed that *PbreOrco* was mainly expressed in antennae, without significant difference in transcription level between males and females (Figure 3). These results are consistent with *Orcos* in *Apolygus lucorum*, *Tenebrio molitor* and *Rhodnius prolixus* (Zhou et al., 2014; Franco et al., 2016; Liu et al., 2016), strongly supporting that *PbreOrco* is essential for insect chemosensation.

Silencing the expression of a targeted gene by RNAi technology is considered as an effective method for functional verification of genes in insects (Huvenne and Smagghe, 2010). To silence a gene through RNAi, direct microinjection and artificial feeding of dsRNA are two frequently-applied approaches. Of them, microinjection is more preferred because it could easily control the precise amount of dsRNA, and induce RNAi more effectively (Franco et al., 2016; Ma et al., 2020). In this study, we also utilized direct injection of dsRNA to introduce RNAi and further study the function of Orco in WSFCs. qRT-PCR results showed that the transcription level of *PbreOrco* was reduced 86.07–90.94% in males and 85.04–87.96% in females after 5–9 days of injection (Figure 4), suggesting that injecting dsRNA is an ideal tool for studies on function of *PbreOrco in vivo*. Although the efficiency of silencing *PbreOrco* was determined to be satisfied within 9 days, the persistence of the silencing effect of other target genes need to be further evaluated. In addition, our results were consistent with that RNAi is a knockdown rather than a knockout method. In WSFCs, however, 9 days was long enough for us to complete the behavioral bioassays. Indeed, the results showed that the silencing of *PbreOrco* sustained for at least 9 days with an effective silencing rate. Furthermore, appropriately increasing amounts of dsRNA injected to insects could potentially prolong the silencing time (Huvenne and Smagghe, 2010; Miller et al., 2012; Joga et al., 2016). In this study, compared with smaller beetles in size (Liu et al., 2016; Zhang et al., 2016; Ma et al., 2020), we injected a relatively higher amounts of dsRNA into WSFCs (3 μg for each beetle) to ensure not only the longer silencing time, but also the silencing efficiency for such a large beetle.

Electrophysiological and behavioral bioassays are conventional approaches to evaluate the potential influences of gene silencing on injected insects (Rebijith et al., 2016; Dong et al., 2017; Bolton et al., 2019). We also employed EAG and Y-tube olfactometer to test the responses of WSFCs to the aggregation pheromone. Compared with dsGFP-injected control, silencing *PbreOrco* almost halved the EAG responses of both female and male WSFCs to 4-methylanisole (Figure 5A) and significantly decreased the preference of WSFCs to 4-methylanisole in Y-tube (Figure 5B). Considering that *PbreOrco* expression was decreased by 90.94% (male) and 87.96% (female) at 7 days of post-injection (Figure 4), it was concluded that the impairment of response to the aggregation pheromone was closely related to the decrease of *PbreOrco* transcript abundance. This indicated that *PbreOrco* was involved in recognition of aggregation pheromone in WSFCs. Furthermore, we carried out cage assays to examine the influences of *PbreOrco* silencing on food-seeking abilities. The results revealed that silencing *PbreOrco* expression directly reduced the response rate and prolonged the response time, indicating that *PbreOrco* silencing significantly impaired olfactory signal-mediated host seeking behavior (Figure 5).

Recent studies documented that silencing Orco simultaneously deteriorated wing differentiation (Fan et al., 2015) and viability (Yang et al., 2016). These unexpected results generally occurred when dsRNA injection was performed at developmental stage (e.g., eggs, larvae, and pupae). The reasons for fewer side effects in injected WSFCs were presumably attributed to the injection at adult stage. Besides affecting olfactory-related behavior, silencing *Orco* at adult stage potentially influences the number of eggs laid, as well as oogenesis and embryogenesis (Libert et al., 2007; Trible et al., 2017; Ma et al., 2020). We did not evaluate the fecundity of injected WSFCs mainly because the life span and oviposition period are too long (>100 days in experimental condition) (Kim et al., 2018) to ensure the decrease of gene transcript abundance.

In summary, we identified the full-length sequence of *PbreOrco* in WSFCs and demonstrated that silencing *PbreOrco* would impair the abilities of WSFCs to detect pheromone and locate food. These results echo the theory about the mechanism of olfactory recognition and are beneficial to development of olfactory-based pest control strategies.

## Data Availability Statement

The original contributions presented in the study are included in the article/Supplementary Material, further inquiries can be directed to the corresponding author/s.

## Author Contributions

TZ designed the research. XZ, PL, QQ, and ML performed the experiments. QQ and RM analyzed the data. XZ, PL, and TZ wrote the manuscript. All authors have read and agreed to the published version of the manuscript.

## Conflict of Interest

The authors declare that the research was conducted in the absence of any commercial or financial relationships that could be construed as a potential conflict of interest.

## Footnotes

1 https://blast.ncbi.nlm.nih.gov/Blast.cgi

2 http://www.cbs.dtu.dk/services/TMHMM/

3 http://www.sacs.ucsf.edu/TOPO-run/wtopo.py

4 http://tree.bio.ed.ac.uk/software/figtree/

## Supplementary Material

Supplementary Figure 1 Electroantennographic (EAG) signal of injected *Protaetia brevitarsis*.

Supplementary Figure 2 The percentage of individuals successfully find food within 20 min.

Supplementary Table 1 Primers used in RNA interference and qRT-PCR.

Supplementary Table 2 Sequences used in sequence alignment and phylogenetic tree.

Supplementary Video 1 Location abilities of injected *Protaetia brevitarsis* to food.
