# Supplementary material for: Characterizing the Role of Orco Gene in Detecting Aggregation Pheromone and Food Resources in Protaetia brevitarsis Leiws (Coleoptera: Scarabaeidae)
Source: Front Physiol. 2021 Apr 13;12:649590. doi: 10.3389/fphys.2021.649590 (PMC8076894; doi:10.3389/fphys.2021.649590)
Supplement: Supplementary Figure 1 — Electroantennographic (EAG) signal of injected Protaetia brevitarsis. [file Data_Sheet_1.docx]

Supplementary Material

**
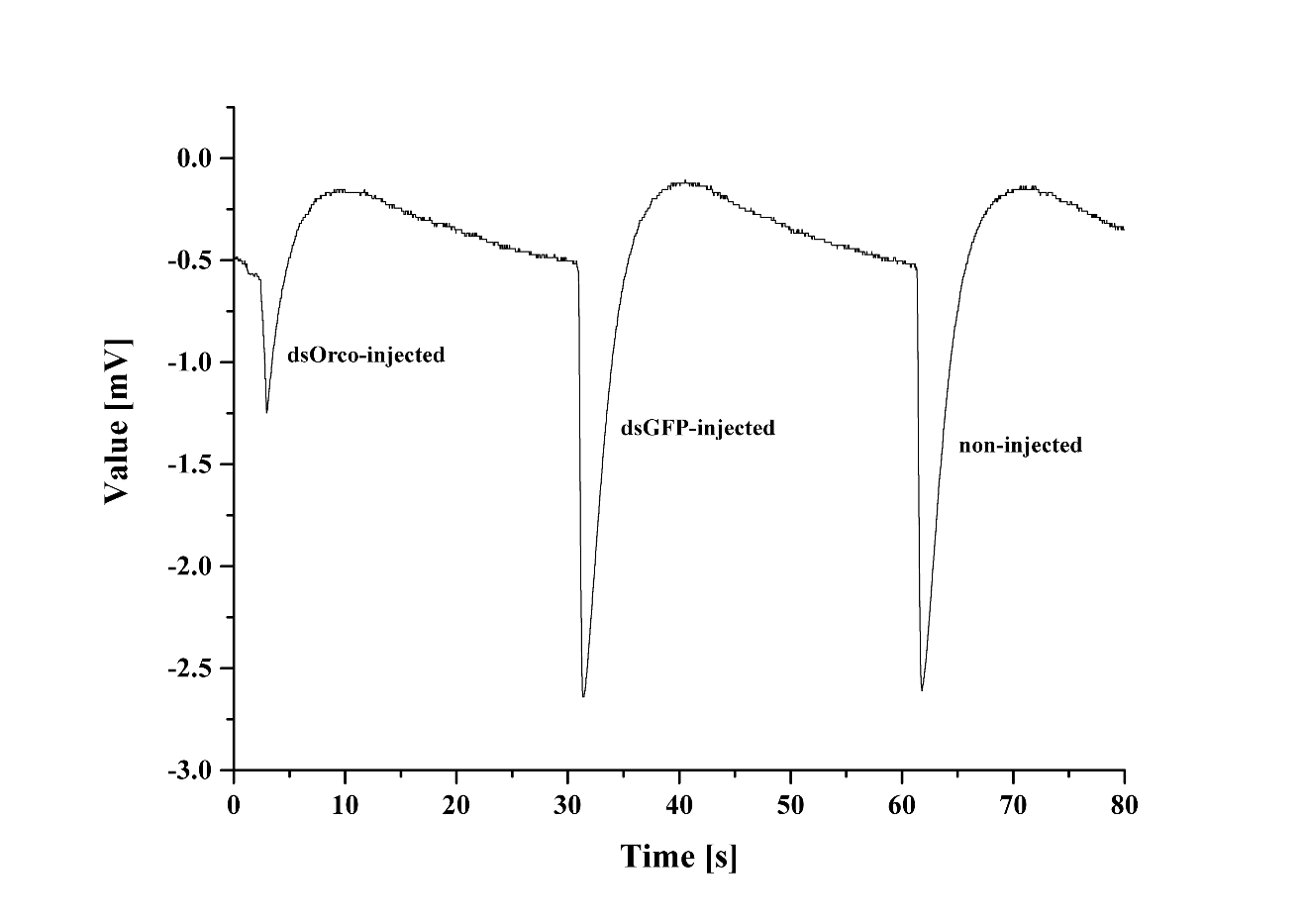
**

**Supplementary Figure S1** Electroantennographic (EAG) signal of injected *Protaetia brevitarsis*.

**
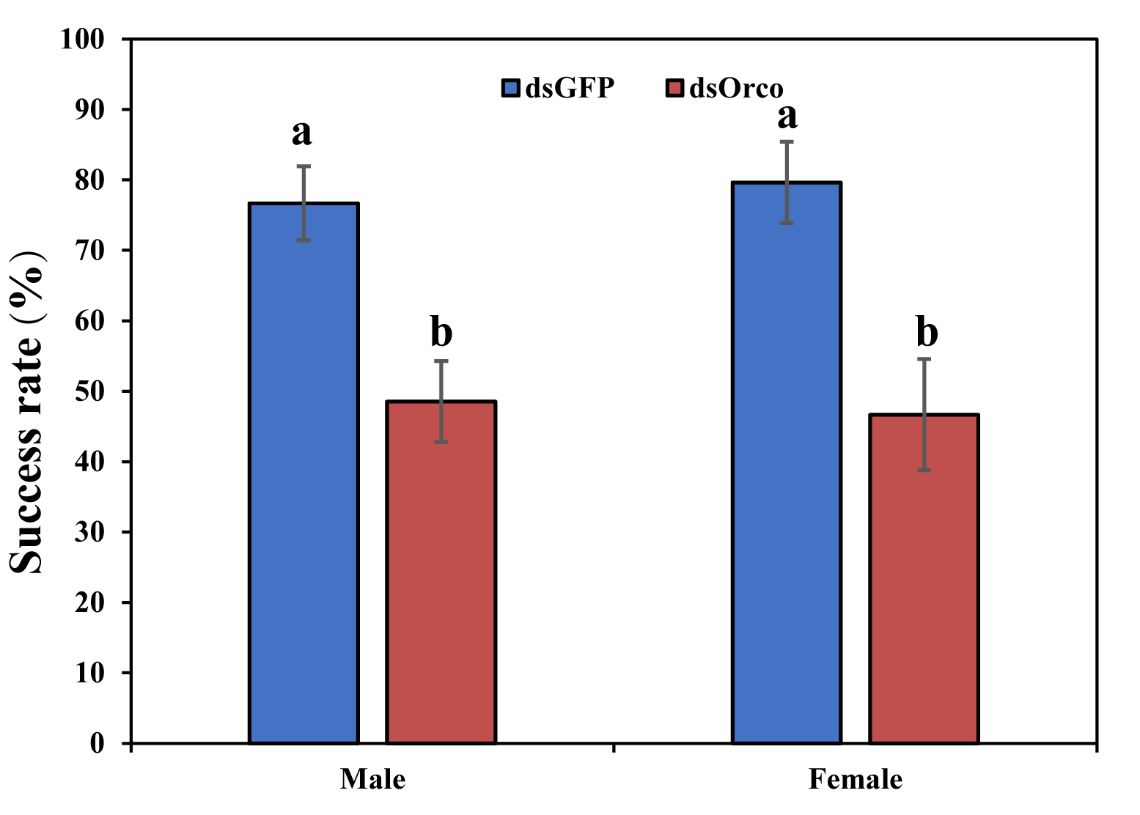
**

**Supplementary Figure S2** The percentage of individuals successfully find food within 20 min.

**Supplementary Table S1**. Primers used in RNA interference and qRT-PCR.

| **Primers' name** | **Sequence (5'-3')** | **Application** |
| --- | --- | --- |
| GSP1 | CTCGGCGCTCTGAATAGGTCTGCAC | 5'RACE |
| GSP2 | CCTTGAGGTGCTGCAATTGCTCGC | 5'RACE |
| PbreOrco-F | ATGATGCAATTCAAGCCC | Full-length validation |
| PbreOrco-R | TCATTTCAATTGTACCAAAACC | Full-length validation |
| dsPbreOrco-F | TAATACGACTCACTATAGGGAGACCAGTTGGATTGCTTGGAC | dsRNA synthesis |
| dsPbreOrco-R | TAATACGACTCACTATAGGGAGAACCACCGCTAAAGTCTTGG | dsRNA synthesis |
| dsGFP-F | TAATACGACTCACTATAGGGAGATGGTCCCAATTCTCGTGGAAC | dsRNA synthesis |
| dsGFP-R | TAATACGACTCACTATAGGGAGACTTGAAGTTGACCTTGATGCC | dsRNA synthesis |
| GADPH2-F | GGCTTTTCGAGACGAACCGT | reference gene |
| GADPH2-R | GCGGCTAAAGCTGTCGGAAA | reference gene |
| PbreORco-RT-F | GGAACCAATCTAATAGTCACC | qRT-PCR |
| PbreORco-RT-R | GGTAGTCCAAGCAATCCAAC | qRT-PCR |

**Supplementary Table S2**. Sequences used in sequence alignment and phylogenetic tree.

>*Protaetia brevitarsis*

MMQFKPQGLVADLMPNIKLIKFSGHFMLNYYADNSGAVHTLRLGFCFAHLFLLLLQYGFTFGNLVKESDDVNDLAANTITVLFFTHCITKFIYFAVRQKLFYRTLGIWNQSNSHPLFLESNNRYHQLALTKMRRLLIVVMVGTMTSWIAWTTITFFGDSVHTRKDPNNENETITEEVPRLLVRSWYPWDAMSGVPYYISLVYQIYYVGFSMLHSNLLDSLFCSWLIFACEQLQHLKEIMKPLMELSATLDTYVPKSADLFRAPSAASRDNLIDNDYNQRNEEATMKGFYTTSQEMGVTYRSGNIQDFSGGIGPNGLTKKQELMVRSAIKYWVERHKHVVRLVTAIGDAYGIALLLHMLTSTITLTLLAYQATKIDGVNKYALTVLGYLIYALAQVFHFCIFGNRLIEESSSVMEAAYSCHWYDGSEEAKTFVQIVCQQCQKAMSISGAKFFTISLDLFASVLGATVTYFMVLVQLK*

>*Holotrichia parallela*

MMQFKPQGLVADLMPNIKLMKFAGHFMLNYYAENSGAVHTLRLGFCFGHLFLMLLQFGFTFGNLVQESDDVNDLAANTITILFFTHCIVKFIYFGVRQKLFYRTLGIWNQSNSHPLFLESNNRYHQLALTKMRRLLIIVMVGTIGSWIAWTTITFLGDSVHTRKDPSNENETITEEIPRLLVRSWYPWDAMSGIPYYITLVYQVYYVGFSMLHSNLLDSLFCSWLIFACEQLQHLKEIMKPLMELSATLDTYVPKSADLFRAPSASSQDRLMDSDYNARNEDVHMKTMYSTHHEMGVTYRSGQLQDFSGGIGPNGLTKKQELMVRSAIKYWVERHKHVVRLVTAIGDAYGIALLLHTSASTITLTLLAYQATKIDGVNKYALTVLGYLFYALTQVFHFCIFGNRLIEESSSVMEAAYSCHWYDGSEEAKTFVQIVCQQCQKAMSISGAKFFTISLVLFASVLGATVTYFMVLVQLK*

>*Holotrichia oblita*

MMKFKPQGLVADLMPNIKLMKFAGHFMLNYYAENSGAVHTLRLGFCFGHLFLMLLQFGFTFGNLVQESDDVNDLAANTITVLFFTHCIVKFIYFGVRQKLFYRTLGIWNQSNSHPLFLESNNRYHQLALTKMRRLLIIVMIGTIGSWIAWTTITFFGDSVHNRKDPNNENETITEEIPRLLIRSWYPWDAMSGIPYYVSLIYQIYYVGFSMLHSNLLDSLFCSWLIFACEQLQHLKEIMKPLMELSATLDTYVPKSADLFRAHSASSQDKLTESDYNARNEDAHMRAMYSTHQEMGVTYRSGQLQEFSSGGIGPNALTKKQELMVRSAIKYWVERHKHVVRLVTAIGDAYGIALLLHMLTSTITLTLLAYQATKIDGVNKYALTVLGYLFYALAQVFHFCIFGNRLIEESSSVMEAAYSCHWYDGSEEVKTFVQIVCQQCQKAMSISGAKFFTISLDLFASVLGATVTYFMVLVQLK*

>*Holotrichia plumbea*

MMQFKPQGLVADLMPNINLMKFAGHFMLNYYSDNGGALHTLRLGFCFGHLFLMLVQFGFTFGNLVQQSDDVNDLAANTITVLFFTHCIVKFIYFGVRQKLFYRTLGIWNQSNSHPLFLESNNRYHQLALTKMRRLLIVVMIGTIGSWIAWTTITFFGDSVHTTKDPNNENETITEEVPRLLIRAWYPWDAMAGIPYYISLVYQIYYVGFSMLHSNLLDSLFCSWLIFASEQLQHLKEIMKPLMELSATLDTYVPKSADLFRAPSASSQDKLTESDYNARNEDAHMRAMYSTHQEMGVTYRSGQLQDFSSGGIGPNGLTKKQELMVRSAIKYWVERHKHVVRLVTAIGDAYGIALLLHMLTSTITLTLLAYQATKIDGVNKYALTVLGYLFYALAQVFHFCIFGNRLIEESSSVMEAAYSCHWYDGSEEAKTFVQIVCQQCQKAMSISGAKFFTISLDLFASVLGATVTHFMVLVQLK*

>*Anomala corpulenta*

MMQFKPQGLVADLIPNIKLMQFSGHFMLNYYAETTGAVHTLRLGFCFGHLFLLLLQFGFTFGNLVQQSDDVNDLAANTITVLFFTHCITKFVYFAVRQKLFYRTLGIWNQSNSHPLFLESNNRYHQLALTKMRRLLIVIMIGTIGSWIAWTTITFFGDSVHTRKDPNNENETITEEVPRLLVRSWYPWDAMSGAAYYVSLVYQIYYVGFSMLHSNLLDSLFCSWLIFACEQLQHLKEIMKPLMELSATLDTYVPKSADLFRAPSASSQDNLVDSDYNQSNEDANLRNLYTTHQEMGVTYRSGNLQEFSSGGIGPNGLSKKQELMVRSAIKYWVERHKHVVRLVTAIGDAYGIALLLHMLTSTIMLTLLAYQATKIDGVNKYALTVIGYLLYALAQVFHFCIFGNRLIEESSSVMEAAYSCHWYDGSEEAKTFVQIVCQQCQKAMSISGAKFFTISLDLFASVLGATVTYFMVLVQLK*

>*Onthophagus taurus*

MMNFKVTGLVADLMPNIRLMQASGHFMLNYYADNNGALHTLRLGYCFMHLFLVLLQYGFTFGNLVQESDDVNDLAANTITVLFFTHCLTKFVYFALRSKLFYRTLGIWNQANSHPLFAESNNRYHALALTKMRRVLAIVVIGTLASWIAWTTITFFGDSTHTRKDPNNENETITEEIPRLLIKSFYPWNAMSGMKYYISLSYQVYYVLFSMLHSNLLDVLFCCWLIFACEQLQHLKEIMKPLMELSATLDTYVPKSADLFRAPSVNSQDNLIDNDALDYNNMKNDELNLKGIYSTHQEMGINYRGGNLQQFDSGGGGIGPNGLTKKQELLVRSAIKYWVERHKHVVRLVTAIGDAYGIALLLHMLTATITLTLLAYQATKIDSVSKYALTVLGYLFYALAQVFLFCIFGNRLIEESSSVMEAAYSCHWYDGSEEAKTFVQIVCQQCQKAMSISGAKFFTISLDLFASVLGATVTYFMVLVQLK*

>*Anoplophora glabripennis*

MMKFKVSGLVADLMPNIRLIQASGHFMFNYHADNSGALHALRLGYSCAHLLFCLFQYGCIFGNLVVEKDDVNYLAANTITVLFFTHCITKFVYFALRSKLFYRTLGIWNQSNSHPLFVESNNRYHALALKKMRTLLICVTATTVLSAAAWTGITFVEESVHNIKDPDNENETITEEIPRLLIKSWYPWDAMSGMAYYGSLIFQIYYVLFSLAHANLMDSLFCSWLIFACEQLQHLKEIMKPLMELSASLDTYVPKSADLFRAPSAKSQDNYIENDYNAKNEELNLKGIYNTRQELGGNFRSGALQTFGQGGVGPNGLTKKQELMVRSAIKYWVERHKHVVRLVTAIGDAYGVALLLHMLTSTVMLTLLAYQATKINGVNTYAATTIGYLVYSLAQVFHFCIFGNRLIEESSSVMEAAYSCHWYDGSEEAKTFVQIVCQQCQKAMQISGAKFFTISLDLFASVLGAVVTYFMVLVQLK*

>*Anoplophora chinensis*

MMKFKVSGLVADLMPNIRLIQASGHFMFNYHADNSGALHALRLGYSCAHLLFCLFQYGCIFGNLVVEKDDVNYLAANTITVLFFTHCITKFVYFALRSKLFYRTLGIWNQSNSHPLFVESNNRYHALALKKMRTLLICVTATTVLSAAAWTGITFVEESVHNIKDPDNENETITEEIPRLLVKSWYPWDAMSGMAYYGSLIFQIYYVLFSLAHANLMDSLFCSWLIFACEQLQHLKEIMKPLMELSASLDTYVPKSADLFRAPSAKSQDNYIENDYNAKNEELNLKGIYNTRQELGGNFRTGALQTFGQGGVGPNGLTKKQELMVRSAIKYWVERHKHVVRLVTAIGDAYGVALLLHMLTSTVMLTLLAYQATKINGVNTYAATTIGYLVYSLAQVFHFCIFGNRLIEESSSVMEAAYSCHWYDGSEEAKTFVQIVCQQCQKAMQISGAKFFTISLDLFASVLGAVVTYFMVLVQLK*

>*Tenebrio molitor*

MMKFKVSGLVADLMPNIRLIQASGHFMLNYHADNSGAVHTLRLGYCIMHLIFMLLQYGCNFVNLIFERGDVNDLAANTITVLFFTHCITKFVYFAARSKLFYRTLGIWNQPNSHPLFVESNNRYHALALKKMRRLLYIIIIWTSFSAIAWTSITFVGDSVHNIKDPDNENMTITEEIPRLLVKAWYPWNAMSGMPYYITLVFQVYYVFFALSHANLLDSLFCSWLIFACEQLQHLKEIMKPLMELSASLDTYVPKSADLFRAPSATSQDNLIENDYNTKNEDLKGVYSTRQELGGHFRGGALQNFGGVGGGVGPNGLTKKQELMVRSAIKYWVERHKHVVRLVTAIGDAYGVALLLHMLTSTIMLTLLAYQATKITGVDKYAATVIGYLLFALAQVFHFCIFGNRLIEESSSVMEAAYSCHWYDGSEEAKTFVQIVCQQCQKAMSISGAKFFTISLDLFASVLGAVVTYFMVLVQLK*

>*Tribolium castaneum*

MMKFKVTGLVADLMPNIRLIQASGHFMLNYHADNSGALHTLRLGYCCMHLVFVLVQYGCNFVNLVLERGDVNDLAANTITVLFFTHCVTKFVYFAVRSKLFYRTLGIWNQPNSHPLFVESNNRYHGIALKKMRRLLYIIIIWTSFSAIAWTGITFVGDSVHNIKDPENENLTITEPIPRLLVKAWYPWDAMSGMPYYITLVFQIYYVFFSLAHANLLDSLFCSWLIFACEQLQHLKEIMKPLMELSATLDTYVPKSADLFRAPSATSQDQLIENDYNEKNEDLKGVYSTRQELGGHFRGGALQNFGSGGVGPNGLTKKQELMVRSAIKYWVERHKHVVRLVTAIGDAYGVALLLHMLTSTIMLTLLAYQATKITGVDKYAATVLGYLLFALAQVFHFCIFGNRLIEESSSVMEAAYSCHWYDGSEEAKTFVQIVCQQCQKAMSISGAKFFTISLDLFASVLGAVVTYFMVLVQLK*

>*Ambrostoma quadriimpressum*

MMKFKVSGLVADLMPNIRLIQASGHFMFNYHADNSGALHALRLGYSCLHLVLCLVQFGCTFGNLVIERNDVNDLAANTITVLFFTHCITKFVYFAVRSKLFYRTLGIWNKANSHPLFLDSNNRYHALSLKKMRTLLICVMTTTILSASAWTAITFVGDSVHNVKDPDNDNETITEEIPRLLIKSWYPWNAMSGTAYYVSVSFQIYYVFFSLAHSNLMDSLFCSWLIFACEQLQHLKEIMKPLMELSASLDTYVPKSADLFRAPSANSQDNLIENEYNEKNEGLNLKGVYNTRQEMGANFRSGALQTFGQGGGGVGPNGLSKKQELMVRSAIKYWVERHKHVVRLVTAIGDAYGVALLLHMLTATVMLTLLAYQATKIDGVNKYAATVIGYLVYSLAQVFHFCIFGNRLIEESSSVMEAAYSCHWYDGSEEAKTFAQIICQQCQKALSISGAKFFTISLDLFASVLGAVVTYFMVLVQLK*

>*Rhynchophorus ferrugineus*

MNTFKVAGLVADLMPNIRLIQASGHFMLNYHADNSGALHGLRLGYCCMHLLFVLLQFGCIFGNLVKEKDNVNDLAANTITILFFTHCLTKFVYFAVRSKLFYRTLGIWNQANSHPIFIESNNRYHALALKKMRNLLYIIMIGTIFSASAWTGITFMGDSVHYIKDPNNENETISEEIPRLLIKSWYPFDAMSGMPYYIALVFQVYYVLFSLLHANLLDSLFCSWLIFACEQLQHLKEIMKPLMELSASLDTYVPKSADLFKAPNSASSQDNLIENEYNSKNDELNLKGVYSTRQELGNLTFRSGALQTFGQGGGGVGPNGLTKKQELMVRSAIKYWVERHKHVVRLVTAIGDAYGVALLLHMLTATIMLTLLAYEATKIDGVNVYAATTIGYLLYSLAQVFHFCIFGNRLIEESSSVMEAAYSCHWYDGSEEAKTFVQIVCQQCQKALSISGAKFFTISLDLFASVLGAVVTYFMVLVQLK*

>*Sitophilus oryzae*

MMNTFKVTGLVADLMPNIRLIQASGHFMLNYHADNSGALHALRLGYPCVHLLFVLLQYGCIFGNLVVEKDNVNDLAANTITILFFTHCLTKFVYFAARSKLFYRTLGIWNQANSHPLFVESNNRYHALALKKMRNLLYIIMIGTIFSAGAWSGITFVGDSVHFVKDPNNENETIPEEIPRLLIKSWYPFNAMSGMSYYIALVFQIYYVLFSLLQANLLDSLFCSWLIFACEQLQHLKEIMKPLMELSASLDTYVPKSADLFKSPSATSHDNLIENDYNPKNDELNLKGVYSTRQELGNLNFRSGALQTFGQGGGGVGPNGLSKKQELMVRSAIKYWVERHKHVVRLVTAIGDAYGVALLLHMLTATIMLTLLAYEATKIDGVNVYAATTLGYLIYSLAQVFHFCIFGNRLIEESSSVMEAAYSCHWYDGSEEAKTFVQIVCQQCQKALSISGAKFFTISLDLFASVLGAVVTYFMVLVQLK*

>*Helicoverpa assulta*

MMTKVKAQGLVSDLMPNIKLMQMAGHFLFNYHSENAGMSNLLRKIYASTHAILIVIHYACMGINMAKYSDEVNELTANTITVLFFAHTIIKLAFFALNSKSFYRTLAVWNQSNSHPLFTESDARYHQIALTKMRRLLYFICGMTVLSVISWVTLTFFGESVRMVTNKETNETLTEVVPRLPLKAWYPFNAMSGTMYIVAFAFQVYWLLFSMAIANLMDVMFCSWLIFACEQLQHLKAIMKPLMELSASLDTYRPNTAELFRASSTEKSEKIPDTVDMDIRGIYSTQQDFGMTLRGAGGRLQNFGQQNPNPNGLTPKQEMLARSAIKYWVERHKHVVRLVASIGDTYGTALLFHMLVSTITLTLLAYQATKINGINVYAFSTIGYLSYTLGQVFHFCIFGNRLIEESSSVMEAAYSCQWYDGSEEAKTFVQIVCQQCQKAMSISGAKFFTVSLDLFASVLGAVVTYFMVLVQLK*

>*Helicoverpa zea*

MTKVKAQGLVSDLMPNIKLMQMAGHFLFNYHSENAGMSNLLRKIYASTHAILIFIHYACMGINMAKYSDEVNELTANTITVLFFAHTIIKLAFFALNSKSFYRTLAVWNQSNSHPLFTESDARYHQIALTKMRRLLYFICGMTVLSVISWVTLTFFGESVRMVTNKETNETLTEVVPRLPLKAWYPFNAMSGTMYIVAFAFQVYWLLFSMAIANLMDVMFCSWLIFACEQLQHLKAIMKPLMELSASLDTYRPNTAELFRASSTEKSEKIPDTVDMDIRGIYSTQQDFGMTLRGAGGRLQNFGQQNPNPNGLTPKQEMLARSAIKYWVERHKHVVRLVASIGDTYGTALLFHMLVSTITLTLLAYQATKINGINVYAFSTIGYLSYTLGQVFHFCIFGNRLIEESSSVMEAAYSCQWYDGSEEAKTFVQIVCQQCQKAMSISGAKFFTVSLDLFASVLGAVVTYFMVLVQLK*

>*Spodoptera littoralis*

MMTKVKAQGLVSDLMPNIKLMQAAGHFLFNYHAENGGMSGLLRKIYASTHAILITIHFACMGINMAQYSDEVNELTANTITVLFFTHTIIKLGFFALNSKSFYRTLAVWNQSNSHPLFTESDARYHQIALTKMRRLLYFICGMTVLSVVSWVTLTFFGESVRLITSKETNETLTEVAPRLPLKAWYPFNAMSGTTYIIAFAFQVYWLLFSMAIANLMDVMFCSWLIFACEQLQHLKAIMKPLMELSASLDTYRPNTAELFRASSTEKSEKIPDTVDMDIRGIYSTQQDFGMTLRGAGGRLQTFGQQNNNPNGLTPKQEMLARSAIKYWVERHKHVVRLVASIGDTYGTALLFHMLVSTITLTLLAYQATKINGINVYAFSTIGYLSYTLGQVFHFCIFGNRLIEESSSVMEAAYSCQWYDGSEEAKTFVQIVCQQCQKAMSISGAKFFTVSLDLFASVLGAVVTYFMVLVQLK*

>*Heliothis virescens*

MMTKVKAQGLVSDLMPNIKLMQMAGHFLFNYHSENAGMSNLLRKIYASTHAILIFIHYACMGINMAKYSDEVNELTANTITVLFFAHTIIKLAFFALNSKSFYRTLAVWNQSNSHPLFTESDARYHQIALTKMRRLLYFICGMTVLSVISWVTLTFFGESVRMITNKETNETLTEVVPRLPLKAWYPFNAMSGTMYIVAFAFQVYWLLFSMAIANLMDVMFCSWLIFACEQLQHLKAIMKPLMELSASLDTYRPNTAELFRASSTEKEKIPDTVDMDIRGIYSTQQDFGMTLRGAGGRLQNFGQQNPNPNGLTPKQEMLARSAIKYWVERHKHVVRLVASIGDTYGTALLFHMLVSTITLTLLAYQATKINGINVYAFSTIGYLSYTLGQVFHFCIFGNRLIEESSSVMEAAYSCQWYDGSEEAKTFVQIVCQQCQKAMSISGAKFFTVSLDLFASVLGAVVTYFMVLVQLK*

>*Bombyx mori*

MMTKVKTQGLVTDLMPCIRLLQAAGHFLFNYHADTSGMNMLLRKIYSSAHAVLIVVHYICMGINMAQYKDEVNELTANTITVLFFAHSIIKLAFFAFNSKSFYRTLAVWNQSNSHPLFTESDARYHQISLSKMRRLLYFICGMTVFSVISWVTLTFFGESVRMIASKETNETLTEPAPRLPLKAWYPFKTMSGGGYVFAFIYQIYFLLFSMALANLLDVIFCSWLIFACEQLQHLKAIMKPLMELSAALDTYRPNTAELFRVSSTDKTEKVPDAVDMDIRGIYSTQQDFGMTLRGAGGKLQNFNAENNPNGLTAKQEMLARSAIKYWVERHKHVVRLVASIGDTYGTALLFHMLVSTITLTLLAYQATKINGINVYAFSTIGYLVYTLGQVFHFCIFGNRLIEESSSVMEAAYSCQWYDGSEEAKTFVQIVCQQCQKAMTISGAKFFNVSLDLFASVLGAVVTYFMVLIQLK*

>*Epiphyas postvittana*

MGKVKTQGLVSDLMPNIKLMQTVGHFLFNYSDETGGMSMLLRKVYASTHAVLIVINFLCMAVNMAQYSDEVNELTANTITVLFFAHTVIKLLFFALNSKNFYRTLAVWNQSNSHPLFTESDARYHQLALNKMRRLLYFIGTVTVMAVVSWITITFFGESVRLIADKESNDTLTEPAPRLPLKAWYPFNAMSGTMYIVAFVYQIYWLLFSMAIANLMDVMFCSWLIFACEQLQHLKAIMKPLMELSASLDTYRPNSSELFRASSTEKSEKVPDPVDLDIRGIYSTQQDFGMMLRGAGGRLQNFNNPNPNNPNGLTQKQEMLARSAIKYWVERHKHVVRLVASIGDTYGTALLFHMLVSTITLTLLAYQATKIDGLNVYAFSTIGYLSYTLGQVFHFCIFGNRLIEESSSVMEAAYSCQWYDGSEEAKTFVQIVCQQCQKAMSISGAKFFTVSLDLFASVLGAVVTYFMVLVQLK*

>*Spodoptera exigua*

MMTKVKAQGLVSDLMPNIKLMQAAGHFLFNYHSENGGMTGLLRKIYASTHAILITIHFACMGINMAQYSDEVNELTANTITVLFFTHTIIKLGFFALNSKSFYRTLAVWNQSNSHPLFTESDARYHQIALTKMRRLLYFICGMTVLSVVCWVALTFFGESVRLITSKETNETLTEVAPRLPLKAWYPFNAMSGTMYIIAFAFQVYWLLFSMAIASLMDVMFCSWLIFACEQLQHLKAIMKPLMELSASLDTYRPNTAELFRASSTEKSEKIPDTVDMDIRGIYSTQQDFGMTLRGAGGRLQNFGQQNNNPNGLTPKQEMLARSAIKYWVERHKHVVRLVASIGDTYGTALLFHMLVSTITLTLLAYQATKINGINVYAFSTIGYLSYTLGQVFHFCIFGNRLIEESSSVMEAAYSCQWYDGSEEAKTFVQIVCQQCQKAMSISGAKFFTVSLDLFASVLGAVVTYFMVLVQLK*

>*Plutella xylostella*

MMNKVKAQGLVSDLMPNIKLMQMAGHFLFNYHEENGGMSMLLRKIYASVHAFLIVIHYLCMLLNMAQYSDDVNELTANTITVLFFAHTVIKLLYFAINSKSFYRTLAVWNQSNSHPLFTESDARYHQLALTKMRRLMYFICAVTVLSVISWVTLTFFGESVRFIPDKETNETLTEPAPRLPLKAWYPFDAMSGGMYIVAFAYQVYWLLFAMAIANLMDVMFCSWLLFACEQLQHLKAIMKPLMELSASLDTYRPNTAELFRANSADKEKVPDPVDMDIRGIYSTQHDFGMTLRGAGGRLQNFGGQQVNNPNGLTQKQEMLARSAIKYWVERHKHVVRLVASIGDTYGTALLFHMLVSTITLTLLAYQATKIDGLNVYAFSTIGYLSYTLGQVFHFCIFGNRLIEESSSVMEAAYSCQWYDGSEEAKTFVQIVCQQCQKAMSISGAKFFTVSLDLFASVLGAVVTYFMVLVQ

>*Conopomorpha sinensis*

MISKGKTVGLASDLMPNIKLMQMAGHFLFNYYDENAGMSMLLRKVYACVHAVLILINFLCMAYNMAKYADDVNELTANTITVLFFVHTIIKLAFFAVNSKSFYRTMAVWNQSNSHPLFAESDARYHQISLSKMRKLLYFICGITGMSVVCWVTITFFGESVRYIFDKETNDTYTEVVPRLPVKAWYPFDAMSGSMYVIAFIYQIYWLLFAMMVANLLDVLFCSWLLFACEQLQHLKDIMKPLIELSASLDTYRPNTAELFRAASAEKEKAPPDSTDLDIRGIYATQHDFGMTLRGAGGRLQTFGQNQNGANPNGLTQKQEMLARSAIKYWVERHKHVVRLVTSIGDSYGTALLFHMLVSTITLTLLAYQATKIDGVNVYAFSTFGYLGYTLGQVFHFCIFGNRLIEESSSVMEAAYSCQWYDGSEEAKTFVQIVCQQCQKPMSISGAKFFTVSLDLFASVLGAVVTYFMVLVQLK*

>*Drosophila melanogaster*

MTTSMQPSKYTGLVADLMPNIRAMKYSGLFMHNFTGGSAFMKKVYSSVHLVFLLMQFTFILVNMALNAEEVNELSGNTITTLFFTHCITKFIYLAVNQKNFYRTLNIWNQVNTHPLFAESDARYHSIALAKMRKLFFLVMLTTVASATAWTTITFFGDSVKMVVDHETNSSIPVEIPRLPIKSFYPWNASHGMFYMISFAFQIYYVLFSMIHSNLCDVMFCSWLIFACEQLQHLKGIMKPLMELSASLDTYRPNSAALFRSLSANSKSELIHNEEKDPGTDMDMSGIYSSKADWGAQFRAPSTLQSFGGNGGGGNGLVNGANPNGLTKKQEMMVRSAIKYWVERHKHVVRLVAAIGDTYGAALLLHMLTSTIKLTLLAYQATKINGVNVYAFTVVGYLGYALAQVFHFCIFGNRLIEESSSVMEAAYSCHWYDGSEEAKTFVQIVCQQCQKAMSISGAKFFTVSLDLFASVLGAVVTYFMVLVQLK*

>*Musca domestica*

MQANLQPTKYTGLVADLMPNIKLMKYSGLFMHAFTGGSALLKNVYSSIHLVLIVLQFIFILVNMALNADEVNELSGNTITALFFTHCITKFVYLAVNQKNFYRTLNIWNQPNSHPLFAESDARYHSIALAKMRKLFFLVMLTTVASAVAWITITFFGESVKFATDKETNSTITVPIPRLPIKSFYPWDASSGMFYMISFGYQAYYLLFSMVHSNLCDVLFCSWLIFACEQLQHLKGIMKPLMELSASLDTYRPNSAALFRSLSANSKSELIQNEEKEPVNDLDMSGIYSTKADWGAQFRAPSTLQTFNGINGGNPNGLTKKQEMMVRSAIKYWVERHKHVVRLVAAIGDTYGAALLLHMLTSTIKLTLLAYQATKITGVNVYAFTVIGYLGYALAQVFHFCIFGNRLIEESSSVMEAAYSCHWYDGSEEAKTFVQIVCQQCQKAMSISGAKFFTVSLDLFASVLGAVVTYFMVLVQLK*

>*Anopheles gambiae*

MQVQPTKYVGLVADLMPNIRLMQASGHFLFRYVTGPILIRKVYSWWTLAMVLIQFFAILGNLATNADDVNELTANTITTLFFTHSVTKFIYFAVNSENFYRTLAIWNQTNTHPLFAESDARYHSIALAKMRKLLVLVMATTVLSVVAWVTITFFGESVKTVLDKATNETYTVDIPRLPIKSWYPWNAMSGPAYIFSFIYQIYFLLFSMVQSNLADVMFCSWLLLACEQLQHLKGIMRSLMELSASLDTYRPNSSQLFRAISAGSKSELIINEEKDPDVKDFDLSGIYSSKADWGAQFRAPSTLQTFDENGRNGNPNGLTRKQEMMVRSAIKYWVERHKHVVRLVSAIGDTYGPALLLHMLTSTIKLTLLAYQATKIDGVNVYGLTVIGYLCYALAQVFLFCIFGNRLIEESSSVMEAAYSCHWYDGSEEAKTFVQIVCQQCQKAMTISGAKFFTVSLDLFASVLGAVVTYFMVLVQLK*

>*Bactrocera dorsalis*

MQPSKYVGLVADLMPNIRLMKYSGLFMHNFTGGSGLFKKIYSSVHLVLVLVQFLLILVNLALNAEEVNELSGNTITVLFFTHSITKFIYLAVSQKNFYRTLNIWNQVNSHPLFAESDARYHAIALAKMRKLFTLVMLTTVASAVAWTTITFFGESVKFAFEKETNSTITVEIPRLPIKSFYPWNAGAGMFYIISFAFQCYYLLFSMVHANLCDVLFCSWLIFACEQLQHLKGIMKPLMELSASLDTYRPNSAALFRSLSANSKSELINNEEKEPTDLDISGVYSSKADWGAQFRAPSTLQTFNGMNGTNPNGLTRKQEMMVRSAIKYWVERHKHVVRLVAAIGDTYGGALLLHMLTSTIMLTLLAYQATKITGVNAYAFTTIGYLGYALAQVFHFCIFGNRLIEESSSVMEAAYSCHWYDGSEEAKTFVQIVCQQCQKAMSISGAKFFTVSLDLFASVLGAVVTYFMVLVQLK*

>*Drosophila pseudoobscura*

MTTTMQPSKYTGLVADLMPNIRAMKYSGLFMHNFTGGSGFMKKVYSSIHLVMLLMQFIFILVNMALNAEEVNELSGNTITALFFTHCITKFIYLAVNQKNFYRTLNIWNQVNSHPLFAESDARYHSIALAKMRKLFFLVMLTTILSATAWTTITFFGDSVKMVVDHETNSSMAVEIPRLPIKSFYPWNAFHGMFYMISFAFQVYYVLFSMIHSNLCDVMFCSWLIFACEQLQHLKGIMKPLMELSASLDTYRPNSAALFRSLSAQSKSELIRNEEKDPGNDLDMSGVYSSKADWGAQFRAPTTLQSFGNGNGNGNGGGAANGANPNGLTKKQEMMVRSAIKYWVERHKHVVRLVAAIGDTYGAALLLHMLTSTIKLTLLAYQATKITGVNVYAFTVVGYLGYALAQVFHFCIFGNRLIEESSSVMEAAYSCHWYDGSEEAKTFVQIVCQQCQKAMSISGAKFFTVSLDLFASVLGAVVTYFMVLVQLK*

>*Apolygus lucorum*

MQKVKMHGLVGDLWPNIRLMQLTGHWLLEYHEENGGMLRLLRMAYCWMTTFSIYIQYAFLVCFLILETYNADEMAAVTITTLFFLHSVTKFTYFAFRSSYFYRTLGAWNQVNSHPLFAESNARHRATALSRMRKLLMIIGTVTILAVFGWTTVTFLDEPVWDKTDPDNVNETISVEIPQLMVYAWYPWDARYGMTYFMTFVFQLYWLFITLAHSNLLDVLFCCFVIFACEQLKHLKEILQPLMELSAALDSVVPNSGDLFKAGSAGSDIALIGNGENGNDFDVRGIYSSQRDFSGFQGGVVNGGTVGPNGLTKRQELLVRSAIKYWVERHKHVVKFVSSIGDTYGSALLLHMLTSTVTLTLLAYQATKIEAVDVYAASTIGYLVYTLGQVFVFCIHGNELIEESSSVMEAAYSCHWYDGSEEAKTFVQIVCQQCQKSLTVSGAKFFTVSLDLFASVFGAVVTYFMVLVQLK*

>*Acyrthosiphon pisum*

MYTFSTNMGYKKDGLIKDLWPNIRLIQLSGLFISEYYDDYSGLAVLFRKIYSWITAIIIYSQFIFIVIFMVTKSNDSDQLAAGVVTTLFFTHSMIKFVYFSTGTKSFYRTLSCWNNTSPHPLFAESHSRFHAKSLSRMRQLLIIVSIVTIFTTISWTTITFFGESVWKVPDPETFNQTMYVPVPRLMLHSWYPWDSSHGLGYIVAFVLQFYWIFITLSHSNLMELLFSSFLVHACEQLQHLKEILNPLIELSATLDSSVHNPAEIFRANSAKNQSINGIDHDYNGSYVNEITEYGTKGENEPNRKGPNNLTSNQEVLVRSAIKYWVERHKHVVKYVSLITECYGSALLFHMLVSTVILTILAYQATKINGVNVFAFSTIGYLMYSFAQIFMFCIHGNELIEESSSVMEAAYGCHWYDGSEEAKTFVQIVCQQCQKPLIVSGAKFFNVSLDLFASVLGAVVTYFMVLVQLK*

>*Lygus lineolaris*

MQKVKMHGLVGDLWPNIRLMQLTGHWLLEYHEETGGMARLLRLAYCWMTTFSVYIQYAFLVCFLILETYNADEMAAVTITTLFFLHSVTKFTYFAFRSKYFYRTLGAWNQVNSHPLFAESNARHRATALSRMRKLLMVIGCVTILAVFSWTTVTFLDDPVWDKTDPDNVNETISVEVPQLMVYAWYPWDAKYGMTYFMTFAFQLYWLFITLAHSNLLDVLFCCFVIFACEQLKHLKEILQPLMELSAALDSVVPNSGDLFKAGSAGSDVALIGNGENGNGNDFDVRGIYSSQRDFSGFQGGITNGGTVGPNGLTKRQELLVRSAIKYWVERHKHVVKFVSSIGDTYGSALLLHMLTSTVTLTLLAYQATKIEGVDVYAASTIGYLVYTLGQVFVFCIHGNELIEESSSVMEAAYSCHWYDGSEEAKTFVQIVCQQCQKSLTVSGAKFFTVSLDLFASVFGAVVTYFMVLVQLK*

>*Lygus hesperus*

MQKVKMHGLVGDLWPNIRLMQLTGHWLLEYHEETGGMARLLRLAYCWMTTFSVYIQYAFLVCFLILETYNADEMAAVTITTLFFLHSVTKFTYFAFRSKYFYRTLGAWNQVNSHPLFAESNARHRATALSRMRKLLMVIGCVTILAVFSWTTVTFLDDPVWDKTDPDNVNETISVEVPQLMVYAWYPWDAKYGMTYFMTFAFQLYWLFITLAHSNLLDVLFCCFVIFACEQLKHLKEILQPLMELSAALDSVVPNSGDLFKAGSAGSDVALIGNGENGNGNDFDVRGIYSSQRDFSGFQGGITNGGTVGPNGLTKRQELLVRSAIKYWVERHKHVVKFVSSIGDTYGSALLLHMLTSTVTLTLLAYQATKIEGVDVYAASTIGYLVYTLGQVFVFCIHGNELIEESSSVMEAAYSCHWYDGSEEAKTFVQIVCQQCQKSLTVSGAKFFTVSLDLFASVFGAVVTYFMVLVQLK*

>*Locusta migratoria*

MQKPHGLVADLWPLIRMVQYSGHWMLEYSGGKALRAIYSSAVSLLVVTQFALMAVNLIQRSGDVNELAANTITVLFFLHPVTKFGYFAVRSKAFYRTLATWNQSNSHPLFAESQARFHQLSVVRMRRLVMYVVSVTALSVVSWTSITFMGDSTREVTDPDNANETITEEVPRLMISTWYPFDASSGMGYMLAFVYQLYWLTATLMHSNLMDVMFCCWLIYACEQLVHLKEIMKPLMELSATLDTVVPHTSELFRAASTLPTNEPLYDAGNGAADGLTIRGIYSSQRDFSGFNRRSAALSTVREADAGGAVSSAGGIGPNGLSKRQEMLVRSAIKYWVERHKHVVRFVGNIGDAYGAALLLHMLTTTVTLTLLAYQATKIDSVDVYAASVLGYLFYTLGQVFLFCVFGNRLIEESSSVMEAAYSCHWYDGSEEAKTFVQIVCQQCQKSLMISGAKFFTVSLDLFASVLGAVVTYFMVLVQLK*

>*Schistocerca gregaria*

MQKPHGLVADLWPLIRMVQYSGHWMLEYSGGLTALRAIYSSVVSVLVVTQFALMAVNPIQRSGDVNELAANTITVLFFLHPITKFAYFAVRSKAFYRTLATWNQSNNHPLFAESQARFHQLSVVRMRRLVMYVVSVTALSVVSWTSITFMGDSTREVADPDNANETITEEVPRLMISTWYPFDASSGMGYMLAFVYQLYWLTATLMHSNLMDVMFCCWLIYACEQLVYLKEIMKPLMELSATLDTVVPHTSELFRAASTLPTNEPLYGMGPDMSNGVTDGMTIRGIYSSQRDFSGFNRRSAALSTVREADSGGAVTSAGGIGPNGLSKRQEMLVRSAIKYWVERHKHVVRFVGNIGDAYGAALLLHMLTTTVTLTLLAYQATKIDSVDVYAASVLGYLFYTLGQVFLFCVFGNRLIEESSSVMEAAYSCHWYDGSEEAKTFVQIVCQQCQ

>*Cephus cinctus*

MMKFKQQGLVADLMPNIRHMQFSGHFMFNYYNDTGGSTKLFHTIYCSIHLFLILLQFGLCCVNLTLERADVDDLTANTITVLFFAHSIIKLAYFAVRSKLFYRTLGIWNNPNSHPLFAESNARYHAIALTKMRRLLAAVGAATILTVCAWTGITFVGDSVKKVTDPVTNETMTVEIPRLMLRSWYPYDASHGMAHVLTLIYQFYFLLITTMDANSLDVLFCSWLLFACEQLQHLKQIMKPLMELSATLDTVVPHTNELFKAGSTDHLRDTQGTQPMAPPPNENMLDMDLRGIYSNRQDFTATFRTAAGMNFNGGVGPNGLTKKQEMLVRSAIKYWVERHKHIVRLVTAIGDAYGVALLFHMLITTVSLTLLAYQATKVNTVDVYAATVIGYVLYTLGQVFLFCIFGNRLIEESSSVMEAAYSCHWYDGSEEAKTFVQIVCQQCQKAMSISGAKFFTVSLDLFASVLGAVVTYFMVLVQLK*

>*Apis mellifera*

MKFKQQGLIADLMPNINLMKATGHFMFNYYTDSSTKHIHKIYCIVHLVLILMQFGFCGINLMMESEDVDDLTANTITMLFFTHSVVKLVYFAVRSKLFYRTLGIWNNPNSHPLFAESNARYHQIAVKKMRILLLAVIGTTVLSAISWTTITFIGDSVKKVIDPVTNETTYVEIPRLMVRSWYPYDPSHGMAHILTLIFQFYWLIFCMADANLLDVLFCSWLLFACEQIQHLKNIMKPLMEFSATLDTVVPNSGELFKAGSAEQPKEQEPLPPVTPPQGENMLDMDLRGIYSNRTDFTTTFRPTAGMTFNGGVGPNGLTKKQEMLVRSAIKYWVERHKHIVRLVTAIGDAYGVALLLHMLTTTITLTLLAYQATKIHAVDTYAASVVGYLLYSLGQVFMLCIFGNRLIEESSSVMEAAYSCHWYDGSEEAKTFVQIVCQQCQKAMSISGAKFFTVSLDLFASVLGAMVTYFMVLVQLK*

>*Nasonia vitripennis*

MMKMKQQGLVADLLPNIRVMQGVGHFMFNYYSEGKKFPHRIYCIVTLLMLLMQYGMMAVNLMMESDDVDDLTANTITMLFFLHPIVKMIYFPVRSKIFYKTLAIWNNPNSHPLFAESNARFHALAITKMRRLLFCVAGATIFSVISWTGITFVDESVKRIVDPETNETTIIPIPRLMIRTFYPFNAMSGAGHVFALIYQFYYLIISMAISNSLDVLFCSWLLFACEQLQHLKAIMKPLMELSATLDTVVPNSGELFKAGSADHLRDSQGVQPSGNGDNVLDVDLRGIYSNRQDFTATFRPTAGTTFNGGVGPNGLTKKQEMLVRSAIKYWVERHKHVVRLVTSVGDAYGVALLLHMLTTTITLTLLAYQATKVNGVNVYAATVIGYLLYTLGQVFLFCIFGNRLIEESSSVMEAAYSCHWYDGSEEAKTFVQIVCQQCQKAMSISGAKFFTVSLDLFASVLGAVVTYFMVLVQLK*

>*Microplitis mediator*

MMKTKHQGLVADLMPNIRLMQISGHFMFNYYGEGKKLMHKIYCSVHLFLILLQFGFVAINLVKEKEDVDDLTANTITILFFLHTLIKIVYFAARSKLFYRTLAIWNNPNSHPLFAESNARYHSIALTKVRRLLFCVGAATVATTISWTTLTFFEDPHVERLNKETNETYIEEIPRLLVRSWYPFDARHGVAHIGMLIYQIYWLFICTVDANSIDVLFCSWLLFACEQLQHLKAIMKPLMELSATLDTVVPNSGELFKAGSADHLRDNDGVPAEPAMNGDNMLDMDLRGIYSNRQDFTATFRPTAGTQYNGGVGPNQLTKKQEMLVRSAIKYWVERHKHIVRLVTAIGDAYGVALLFHMLITTITLTLLAYQATKVNGVNVYAASTIGYLLYSLGQVFLFCIFGNRLIEESSSVMEAAYSCHWYDGSEEAKTFVQIVCQQCQKAMSISGAKFFTVSLDLFASVLGAVVTYFMVLVQLK*
